# Supplementary feeding increases nestling feather corticosterone early in the breeding season in house sparrows

**DOI:** 10.1002/ece3.3114

**Published:** 2017-06-30

**Authors:** Noraine Salleh Hudin, Liesbeth De Neve, Diederik Strubbe, Graham D. Fairhurst, Carl Vangestel, Will J. Peach, Luc Lens

**Affiliations:** ^1^ Terrestrial Ecology Unit Department of Biology Ghent University Ghent Belgium; ^2^ Department of Biological Sciences Faculty of Science & Mathematics Universiti Pendidikan Sultan Idris Tanjong Malim Perak Malaysia; ^3^ Department of Biology University of Saskatchewan Saskatoon Saskatchewan Canada; ^4^ Joint Experimental Molecular Unit Royal Belgian Institute of Natural Sciences Brussels Belgium; ^5^ RSPB Centre for Conservation Science RSPB Sandy Bedfordshire UK

**Keywords:** altricial bird, body condition, chronic stress, fluctuating asymmetry, food supplementation, laying date

## Abstract

Several studies on birds have proposed that a lack of invertebrate prey in urbanized areas could be the main cause for generally lower levels of breeding success compared to rural habitats. Previous work on house sparrows *Passer domesticus* found that supplemental feeding in urbanized areas increased breeding success but did not contribute to population growth. Here, we hypothesize that supplementary feeding allows house sparrows to achieve higher breeding success but at the cost of lower nestling quality. As abundant food supplies may permit both high‐ and low‐quality nestlings to survive, we also predict that within‐brood variation in proxies of nestling quality would be larger for supplemental food broods than for unfed broods. As proxies of nestling quality, we considered feather corticosterone (CORT_f_), body condition (scaled mass index, SMI), and tarsus‐based fluctuating asymmetry (FA). Our hypothesis was only partially supported as we did not find an overall effect of food supplementation on FA or SMI. Rather, food supplementation affected nestling phenotype only early in the breeding season in terms of elevated CORT_f_ levels and a tendency for more variable within‐brood CORT_f_ and FA. Early food supplemented nests therefore seemed to include at least some nestlings that faced increased stressors during development, possibly due to harsher environmental (e.g., related to food and temperature) conditions early in the breeding season that would increase sibling competition, especially in larger broods. The fact that CORT_f_ was positively, rather than inversely, related to nestling SMI further suggests that factors influencing CORT_f_ and SMI are likely operating over different periods or, alternatively, that nestlings in good nutritional condition also invest in high‐quality feathers.

## 1 INTRODUCTION

Coping with environmental perturbations, such as bad weather, predators, or food shortages, is a primary challenge faced by organisms. These unpredictable stressors often create energetically demanding conditions that may trigger an acute response in the form of short‐term elevations of the hormone corticosterone (CORT; the end product of activation of the hypothalamic–pituitary–adrenal axis). CORT redirects energy expenditure by changing physiology and behavior in order to meet such demands and to return to normal activities (referred to as “allostatic load” (McEwen & Wingfield, 2003; Romero, 2004)). Acute stress responses are considered adaptive as they enable individuals to cope with perturbations and increase immediate survival, as well as to prepare them to successfully respond to the reoccurrence of stressors (Blas, Bortolotti, Tella, Baos, & Marchant, 2007; Romero, 2004; Sapolsky, Romero, & Munck, 2000). Along these lines, Egyptian vultures *Neophron percnopterus* elevated their CORT levels when faced with limited food resources, which increased their foraging activity to avoid starvation (Carrete et al., 2013). However, when individuals coping with stressors fail to improve their condition through behavioral and/or physiological modifications, they may experience chronically elevated CORT levels (Kitaysky, Kitaiskaia, Piatt, & Wingfield, 2003; Sapolsky et al., 2000) which can lead to detrimental effects on fitness (Hayward & Wingfield, 2004; Kitaysky et al., 2003; Pravosudov & Kitaysky, 2006; Rubolini et al., 2005). Chronic stress may be particularly harmful during early life stages because young animals are typically vulnerable to adverse conditions. A substantial body of evidence suggests that elevated CORT levels during prenatal or nestling development affect growth and can weaken immune function, with long‐term consequences for physiology, morphology, and behavior (Bebus, Small, Jones, Elderbrock, & Schoech, 2016; Blas et al., 2007; Kitaysky et al., 2003; Love & Williams, 2008; Rubolini et al., 2005; Saino, Romano, Ferrari, Martinelli, & Møller, 2005; Schmidt, Moore, MacDougall‐Shackleton, & MacDougall‐Shackleton, 2013; Schoech, Rensel, & Heiss, 2011; Spencer, Evans, & Monaghan, 2009; Spencer & Verhulst, 2007). Hence, exposure to periods of stress at the nestling stage can have an impact on fitness and may therefore translate into population level effects.

Limited food availability is an important early‐life stressor known to cause elevated CORT levels (Boonstra, 2013; Herring, Cook, Gawlik, & Call, 2011; Pravosudov & Kitaysky, 2006; Saino, Suffritti, Martinelli, Rubolini, & Møller, 2003), especially in altricial bird species in which starvation is known to be the main cause of nestling mortality (Martin, 1987). Nestlings may respond to nutritional restriction by increasing begging displays (Kitaysky, Wingfield, & Piatt, 2001a; Loiseau, Fellous, Haussy, Chastel, & Sorci, 2008b) to elicit an increase in parental provisioning rates (Roulin & Dreiss, 2012). Prolonged periods of limited food supplies may also increase sibling competition and result in brood reduction where high‐quality nestlings survive and gain more food from their parents (Roulin & Dreiss, 2012).

Reduced food provisioning of nestlings may arise when environmental conditions are unfavorable for invertebrates, which constitute the most important source of proteins in the diet of passerine nestlings (White, 2008). A reduction in invertebrate abundance and diversity may, for example, occur due to urbanization (Vergnes, Pellissier, Lemperiere, Rollard, & Clergeau, 2014), and studies of birds have already proposed a lack of invertebrate prey as a primary cause of lower observed breeding success in urban areas compared to less urbanized areas (Chamberlain et al., 2009; Mennechez & Clergeau, 2006). House sparrows (*Passer domesticus*) have been associated with humans for hundreds of years and are a classical example of an urban exploiter (e.g., Kark, Iwaniuk, Schalimtzek, & Banker, 2007). Yet, sparrow populations in urban centers across Europe started to decline in the late 1980s (De Laet & Summers‐Smith, 2007; Shaw, Chamberlain, & Evans, 2008). Several hypotheses have been proposed to explain this decline, among which are a loss of nesting sites and adequate food sources (Summers‐Smith, 2003). In support of this hypothesis, fledging production was increased when house sparrow nests were supplemented with mealworms in domestic gardens in the city of Leicester, UK (Table S1 *sensu* Peach, Sheehan, & Kirby, 2014). Similarly, a significant positive influence of mealworm supplementation on per‐capita fledgling counts was shown in house sparrows from suburban areas in London (Peach, Mallord, Ockendon, Orsman, & Haines, 2015), where a population decline of 60% was observed during the decade preceding the study (Raven, Noble, & Baillie, 2007). However, despite the improved reproductive success resulting from the experiment, house sparrows did not show population growth or recovery (Peach et al., 2015). These observations suggest that postfledging mortality may have been high, which could be linked to reduced phenotypic and/or physiological quality arising during the nestling period.

So far, avian food supplementation studies during the breeding season mainly evaluated outcomes on maternal investment (e.g., laying date, incubation, clutch, and egg size) and reproductive success (number of fledglings) (Ruffino, Salo, Koivisto, Banks, & Korpimaki, 2014), but there is a surprising lack of information on the potential effects on the quality of fledglings. In this study, we explore multiple proxies of fledgling quality collected as part of the food supplementation experiment performed by Peach et al. (2014). This study demonstrated that daily provision of live invertebrate prey increased reproductive success (fledgling production) by 55% but had no significant impact on average nestling tarsus length or body mass (Table S1 *sensu* Peach et al., 2014). In line with these findings, we hypothesize that under experimental food supplementation, parents could fulfill the basic nutritional requirements of more nestlings, allowing low‐quality nestlings to survive instead of dying from starvation in the completion for food with higher‐quality nestlings. If so, food supplemented nests are expected to contain both high‐ and low‐quality nestlings at the end of the nestling period, resulting in no effect on average nestling quality, but showing larger within‐brood variation in proxies of nestling quality compared to nests without access to supplemental food.

To test our hypothesis, we used three indices of nestling quality collected from the birds in the study reported by Peach et al. (2014): CORT levels in tail feather samples (feather corticosterone, CORT_f_), fluctuating asymmetry (FA) of tarsus length, and scaled mass index (SMI). Given that feathers start to emerge soon during nestling development (i.e., day 4, Anderson, 2006) and newly grown feathers incorporate circulating CORT (Fairhurst, Marchant, Soos, Machin, & Clark, 2013; Jenni‐Eiermann, Helfenstein, Vallat, Glauser, & Jenni, 2015), CORT_f_ provides an integrated signal of adrenocortical activity (i.e., both baseline and temporary acute elevations) in response to allostatic demands during the nestling period (Fairhurst et al., 2013; Will et al., 2014; see Romero & Fairhurst, 2016 for a review). FA is the within‐individual difference in size of bilaterally symmetrical traits and, as an indicator of developmental stress, higher FA is indicative for a lower ability to buffer against stressors during development (Auffray, Renaud, Alibert, & Nevo, 1999). SMI is a good proxy of nestling body condition (Peig & Green, 2009), which typically shows a positive correlation with recruitment probability in passerine bird species (Cleasby, Nakagawa, Gillespie, & Burke, 2010; Monrós, Belda, & Barba, 2002).

## 2 MATERIAL AND METHODS

### 2.1 Species and study sites

House sparrows breed from April until August, during which pairs can produce up to four clutches (Summers‐Smith, 1988). House sparrows lay clutches of typically 3–5 eggs, with incubation lasting approximately 11 days, and with chicks fledging around 14 days after eggs hatch (Summers‐Smith, 1988). Wing feathers typically first emerge in 4‐day‐old chicks after which feather growth is approximately linear until fledging (Anderson, 2006). Sparrow nestlings are fed almost exclusively on invertebrates such as beetles, caterpillars, and aphids, but as nestlings get older, more vegetable material is added to their diet (Anderson, 2006). Nutritional stress affecting nestling house sparrows is probably caused by a lack of suitable invertebrate prey especially larger items such as caterpillars and beetles, resulting in low sparrow nestling body mass (Peach, Vincent, Fowler, & Grice, 2008) and smaller body size in adult house sparrows in urban areas (Seress et al., 2012).

We conducted this study on house sparrow nestlings raised in nest boxes in three rural (Houghton‐on‐the‐Hill, Hungarton, and Keyham) and three suburban (Braunstone, Thurmaston, and Western Park) sites in Leicester, UK, in 2008 (Table A1 in Peach et al., 2014). Nest boxes (all single chamber) were fixed to the sides of mainly residential buildings (usually 2–4 boxes per property), 4–5 m above ground level. Each occupied nest box was categorized as being situated in either suburban or rural localities depending on the presence of farmland within 100 m of the box (present = rural, absent = suburban). Data for this study were obtained from 29 nests situated in rural areas and from 16 nests in suburban areas.

### 2.2 Supplementary feeding experiment

Supplementary feeding of mealworms (*Tenebrio molitar*) was initiated at 10 garden locations spread across all six study sites between mid‐ and late April. Feeding always began after nesting had started (a full clutch was usually laid) but before any eggs hatched, and continued on a daily basis until early August when nesting ceased. The supplementary feeding, therefore, had the potential to influence a range of reproductive parameters but not the size or timing of first clutches. In total, 12 of 29 and five of 16 nests in rural and suburban areas, respectively, had access to the supplementary feeding. At each feeding location, a total of 33 g of live mealworms (comprising approximately 300 mixed‐size worms) were provided each day, split evenly between early morning and late afternoon feeds. Worms were provided in a single metal feeder comprising a holding tray, a protective rainfall cover and a surrounding metal cage to exclude larger birds like European starlings (*Sturnus vulgaris*).

All feeders were placed in private gardens within 30 m of a nest box occupied by breeding house sparrows. Nest box observations were carried out to determine whether mealworms were being provided to the chicks (see Peach et al., 2014 for further details). Although we did not measure casual provisioning of supplementary food by local people, no residents with occupied nest boxes (or their immediate neighbors) provided mealworms or any other form of protein. There were also no retail outlets or fast‐food outlets within 100 m of any nest so that potential food sources of waste were not available. Provisioning of seed and vegetable material (mainly bread) was widespread and probably ubiquitous across our study areas but is unlikely to have been confounded with our feeding treatment. Habitat composition was similar in fed and unfed localities (Peach et al., 2014) suggesting that the availability of key foraging habitats was also unlikely to be confounded with our feeding treatment.

The contents of all nest boxes containing fresh nesting material were checked at least once each week between April until late mid‐August to determine the laying date (LD; 1 March equals to LD = 1) and number of eggs and chicks. The number of live chicks present at 9–13 days after hatching was presumed to be the number of fledglings. At that time, nestlings were weighed (mass to the nearest 0.1 g) and three independent measurements of the left and right tarsus length were taken of every nestling (to the nearest 0.1 mm) for analysis of FA. From each nestling, two tail feathers were collected and kept in individual envelopes for CORT_f_ analysis.

### 2.3 CORT analysis

We used a methanol‐based procedure (Bortolotti, Marchant, Blas, & German, 2008) to extract CORT from feathers in two batches. After removing the calamus from each feather, we cut the remaining samples into small pieces (<5 mm^2^) and added 10 ml of methanol (HPLC grade, VWR International, Mississauga, Ontario, Canada) to each sample. We then sonicated samples in a water bath at room temperature for 30 min, followed by overnight incubation in a water bath at 50°C. We separated methanol from the feather pieces using vacuum filtration and placed the methanol extracts in a 50°C water bath to evaporate in a fume hood. Once extracts were dry, we reconstituted them in a small volume of phosphate‐buffered saline (PBS; 0.05 m, pH 7.6) and stored them at −20°C until analysis by radioimmunoassay (RIA). We assessed the efficiency of the extraction procedure by including three feather samples spiked with a small amount (approximately 5,000 CPM) of ^3^H‐labeled corticosterone (see Appendix S1 in Bortolotti et al. (2008) for more details). On average, 91% (*SD* = 1) of the radioactivity was recoverable in the reconstituted samples, and CORT values were adjusted for recoveries.

We analyzed CORT by RIA as in previous studies (Bortolotti et al., 2008; Fairhurst et al., 2013), and this technique has been replicated in house sparrows (Treen, Hobson, Marchant, & Bortolotti, 2015). We assayed duplicates of reconstituted methanol extracts in three assays using a commercial antiserum (Sigma‐Aldrich, St. Louis, MO, USA; product# C8784). Serial dilutions of sample extracts were parallel to the standard curve, indicating no interference with the antibody. We computed assay variation using three aliquots, each measured in duplicate, of the same standard CORT solution, created from purified CORT (Sigma‐Aldrich), in each assay. Average intra‐assay coefficient of variation (CV) was 8.5% (*SD* = 1.5), and interassay CV was 11.1%. The average detection limit of our assays was 14.20 pg CORT/100 μl sample (*SD* = 4.01), and all values were above detection limits. Data values were normalized by length (i.e., pg CORT/mm of feather) to correct for the time‐dependent deposition of CORT (Bortolotti, 2010; Jenni‐Eiermann et al., 2015; see Romero & Fairhurst, 2016 for a discussion). CORT_f_ assays were performed at the University of Saskatchewan, Canada.

### 2.4 Measurement of fluctuating asymmetry (FA)

Levels of environmental and nutritional stress between nestlings of broods with and without access to supplementary feeding were assessed through measurements of FA of tarsus length. As FA estimation was based on repeated measurements of left and right trait sides, we first estimated the level of repeatability among repeated measurements within each side. Based on a one‐way ANOVA of the within‐ and between‐side mean squares (Lessells & Boag, 1987), the repeated measurements of tarsus length showed very high statistical repeatability (right tarsus: *r* = .997, *n* = 83, *p* < .001; left tarsus: *r* = .992, *n* = 83, *p* < .001). Despite this high repeatability, the fact that the degree of FA is often very small (typically on the order of ≤1% of the size of the trait; Møller & Swaddle, 1997), measurement error (ME) may still cause an upward bias in the between‐sides variance if not appropriately corrected for (Merilä & Björklund, 1995; Palmer & Strobeck, 1986; Van Dongen, Molenberghs, & Matthysen, 1999). Therefore, tarsus FA was analyzed through mixed‐regression analysis with restricted maximum‐likelihood (REML) parameter estimation (Van Dongen et al., 1999). While yielding identical FA estimates as two‐way mixed ANOVA models, the REML method allows to test for FA significance, to model heterogeneity in FA and measurement error among populations or treatments, to test for directional asymmetry (DA), and to obtain estimates of individual FA that are unbiased with respect to ME and DA. Fixed intercepts estimate overall trait size, fixed slopes estimate DA, and the random intercepts and slopes (both estimated *within* individuals) estimate the variation in individual trait value and individual FA, respectively (Van Dongen et al., 1999). Variance in signed FA was much larger than variance in ME and was highly significant (likelihood‐ratio test: *p* < .0001). FA measurements were not biased by directional asymmetry after correcting the denominator degrees of freedom by Satterthwaite's formula (Verbeke & Molenberghs, 1997). For hypothesis testing, unbiased FA values per individual were calculated as the variance components of the slopes of the individual regression lines in the mixed‐regression model.

### 2.5 Scaled mass index

As an estimate of body condition, we used the scaled mass index (SMI; Peig & Green, 2009), which adjusts the mass of all individuals to that which they would have obtained if they had the same body size. SMI was calculated using the equation of the linear regression of log‐body mass on log‐tarsus length estimated by type 2 (standardized major axis; SMA) regression (Peig & Green, 2009). Following Bókony, Seress, Nagy, Lendvai, and Liker (2012), we first verified whether tarsus length was most strongly correlated with body mass on a log–log scale (*r* = .78, *p* < .001) and consequently applied that variable to scale body mass. No outliers were present in the data (i.e., all |standardized residual| < 3, max value was 2.73), the regression slope was 2.95, and average tarsus length was 20.2 mm. We thus calculated the SMI as body mass × (20.2/tarsus length)^2.95^ (Peig & Green, 2009, 2010).

### 2.6 Statistical analysis

We first used univariate tests to explore how our three indicators of nestling quality (i.e., CORT_f_ levels, tarsus‐based FA, and SMI) related to each other, before building three separate linear mixed models (lmer function of R library ‘lme4’; Bates et al., 2015) to assess how these indicators relate to the experimental provisioning of food (fed or not), taking into account factors that could affect our proxies of nestling quality, such as laying date, brood size, urbanization (rural vs. suburban), nestling age (days), and brood reduction. Brood reduction was calculated as the difference between the number of hatched eggs and fledged young. Hence, it measures the number of young that died in the nest and is interpreted as a measure of intensity of food competition among siblings before brood reduction takes place (Mock & Parker, 1997; Soler & Aviles, 2010). We started with models that included all variables mentioned above. SMI, CORT_f_, and FA are three physically unrelated metrics that may relate to stressful conditions during the nestling period via different mechanisms. In order to explain the variation that did not covary between these parameters, we added the remaining metrics as covariates to each model. Correlations between fixed continuous predictors were weak (all *r* < .44). To test whether effects of experimental food provisioning changed over the breeding season or differed between urban and rural sites, the two‐way and three‐way interactions between laying date, urbanization and experimental food provisioning were also added to the models. A nested random effect (i.e., nest box within study site) was included to account for the nonindependence of nestlings from the same nest and of nest boxes within the same study site, respectively.

During hypothesis testing, we adopted a frequentist approach whereby full models (i.e., models containing all explanatory variables considered above) were reduced in a stepwise manner, by excluding the variable with the highest *p*‐value until only predictors with *p* < .05 remained. Statistics and *p*‐values mentioned in the text and table are invariably referring to the final model (i.e., only significant terms included), whereas statistics and *p*‐values of nonsignificant terms were obtained by fitting each nonsignificant term separately into the minimal model. As Larsen et al. (2015) showed a U‐shaped relationship of CORT levels with timing of breeding, CORT_f_ models were run with laying date both as a linear and a quadratic effect. Likelihood‐ratio tests were used to assess whether CORT_f_ levels were best explained by quadratic or by linear terms, using a chi‐square test (R function ‘ANOVA’) to select the best fitting minimal model. When treating laying date as a categorical variable (early vs. late breeding), however, similar results were obtained (data not shown).

Lastly, to test whether nests provided with supplemental food exhibited a higher variation in stress levels among nestlings, we first calculated the difference between the young with the highest and the lowest value of CORT_f_, tarsus‐based FA and SMI in each nest. This range value was then used as the dependent variable in linear mixed model with the same fixed effects as explained above (study site was included as a random effect). For this analysis, we could only include nests for which all mentioned variables could be collected for at least two juveniles, reducing the sample size from 45 to 33 nests.

## 3 RESULTS

The three indicators of nestling quality were positively correlated with one another (CORT_f_ levels vs. tarsus‐based FA: *r *= .23, *p* = .038; CORT_f_ levels vs. SMI: *r *= .27, *p* = .016; SMI vs. tarsus‐based FA: *r *= .34, *p* = .0017).

**Figure 1:**
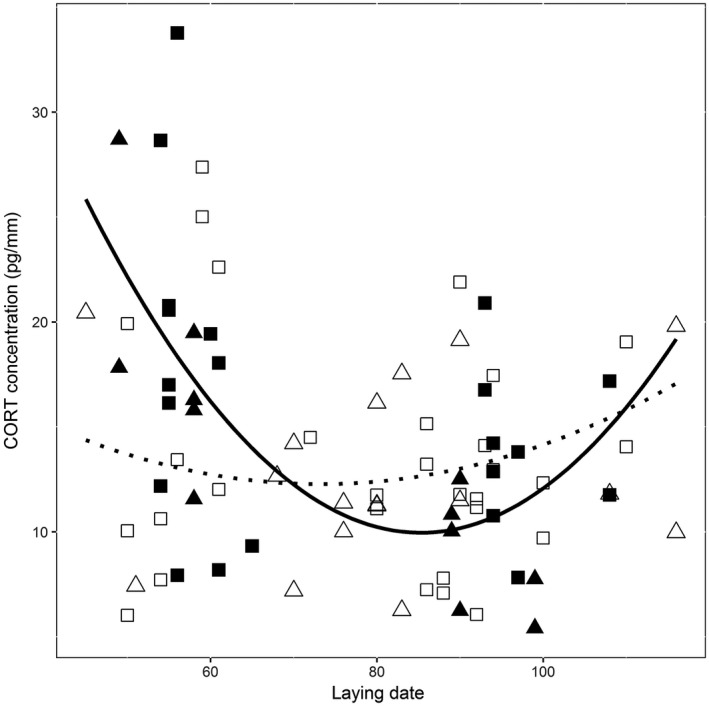
Relationship between laying date and feather corticosterone (CORT_f_) levels measured in sparrow nestlings from rural (rectangles) and suburban (triangles) areas. Nest boxes with access to supplemental food are filled black. The solid line indicates the trend in CORT_f_ levels throughout the breeding season for nests with access to supplementary food, while the dotted line depicts the trend for nests without such access (full model information in Table S1)

When assessing variation in CORT_f_ levels among nestlings, a model including a quadratic laying date term provided a better fit than a linear model (χ^2^(2) = 7.42, *p* = .024). The quadratic model included a significant interaction between laying date and access to experimentally provided supplemental food, as well as significant positive effects of brood reduction and SMI (Table 1). CORT_f_ levels were highest early and late in the breeding season (*F*_2,72_ = 5.70, *p* = .0051; Figure 1), and early in the breeding season nestlings with access to supplemental food were characterized by larger CORT_f_ concentrations than unsupplemented nestlings (laying date * food provisioning: *F*_2,72_ = 5.34, *p* = .0069; Table S2, Figure 1). In addition, CORT_f_ levels were higher for nestlings with larger SMI and for those originating from nests where brood reduction took place (estimate and standard error = 0.60 ± 0.27, *t* = 2.26, *p* = .027; 1.66 ± 0.64, *t* = 2.59, *p* = .012, respectively; Table 1). Other included variables failed to reach statistical significance (i.e., all *p* > .29, see Table S2). FA was higher for heavier nestlings (0.024 ± 0.0073, *t* = 3.33, *p* = .0014) and tended to be lower later in the breeding season (−0.0017 ± 0.0009, *t* = 1.88, *p* = .063; Table S3). Besides the relationship with FA (4.19 ± 1.24, *t* = 3.37, *p* = .0014), nestling SMI declined throughout the breeding season (−0.039 ± 0.015, *t* = −2.58, *p* = .013; Table S4).

**Table 1:** Results from reduced general linear mixed models explaining variation in feather corticosterone (CORT_f_) levels, tarsus‐based FA, and SMI among house sparrow nestlings. Initial models included food provisioning (fed vs. unfed), laying date (linear and quadratic terms), brood size, brood reduction, nestling age (days), and urbanization (rural vs. suburban). Only significant terms are listed here, and full model results are given in Tables S1–S3

Dependent variable	Predictor variable	Estimate	*SE*	*df*	*t*	*p*
CORT_f_	Laying date (linear term)	−25.140	8.004	72	−3.141	.0020
	Laying date (quadratic term)	28.781	12.497	72	2.303	.0240
	Brood reduction	1.658	0.640	72	2.590	.0120
	SMI	0.604	0.267	72	2.261	.0270
	Food provisioning	−0.919	−0.919	72	−0.781	.437
	Laying date (linear term)*food provisioning	29.398	10.855	72	2.708	.0080
FA	SMI	0.024	0.0073	73.850	3.330	.0014
SMI	Laying date (linear term)	−0.036	0.0150	41.980	−2.582	.0130
	FA	4.192	1.2430	50.850	3.373	.0014

Supplemented nests exhibited higher among‐nestling variation in both CORT_f_ and FA at the beginning of the breeding season (Tables S5 and S6), while the range in indicators of nestling quality did not vary with laying date for control nests. However, these patterns failed to reach statistical significance, possibly because of the limited sample size (CORT_f_: interaction between laying date and food provisioning *F*_1,27_ = 1.862, *p* = .074; tarsus‐based FA: *F*_1,27_ = 2.298, *p* = .14; see Tables S5 and S6). No such seasonal pattern was apparent for SMI measurements (*F*_1,27.35_ = 0.170, *p* = .684; see Table S7).

## 4 DISCUSSION

Given that our experimental food supplementation resulted in higher fledging production (Table S1 *sensu* Peach et al., 2014) but not, in a separate study, enhanced population growth (Peach et al., 2015), we hypothesized here that food supplementation allows parents to fulfill the requirements of both low‐ and high‐quality nestlings. As such, we predicted an increase in within‐nest variation in nestling quality, but not in average quality, in supplemented nests. These predictions were partially supported by our results, as we did not find an overall effect of food supplementation on CORT_f_, tarsus‐based FA or nestling body condition, while there was a slight, not significant, tendency of higher within‐nest variation in CORT_f_ and tarsus‐based FA early in the breeding season, but not SMI.

Yet, we also found that food supplementation affected nestling phenotype in terms of elevated CORT_f_ levels (controlled for SMI, Table 1), but only early in the breeding season. So early food supplemented nests seemed to include at least some nestlings that faced increased stressors during development. A possible explanation for this early breeding season effect could be that the harsher weather conditions of early spring produced suboptimal conditions, in terms of microclimate and food availability (i.e., insects), which could increase sibling competition (e.g., for food or for a warm place in the nest box), as both of these factors have been shown to affect allostatic load resulting in higher CORT concentrations (Braasch, Becker, & Groothuis, 2014; Fairhurst, Treen, Clark, & Bortolotti, 2012; Kitaysky, Kitaiskaia, Wingfield, & Piatt, 2001b; Lopez‐Jimenez et al., 2016). Such an effect of sibling competition, if present, should be most apparent in food supplemented nests, because more nestlings survived (Table S1 *sensu* Peach et al., 2014), and elevated CORT_f_ levels are expected to be most marked in lowest ranked nestlings (e.g., Lopez‐Jimenez et al., 2016). This may explain the tendency for within‐nest variation in CORT_f_ and tarsus‐based FA to be greater in supplementary fed nests early in the breeding season. The fact that nestlings also exhibited higher average CORT_f_ levels in nests that suffered brood reduction further supports the idea that sibling competition may increase allostatic load in house sparrows.

Unexpectedly, the three indices used in this study to measure nestling quality were positively correlated, regardless of the experimental treatment. Nestlings in good body condition (SMI) were also those with higher levels of CORT_f_ and higher tarsus‐based FA. Rather, we expected that elevated levels of glucocorticoids during development, reflected in high levels of CORT_f_ (Bortolotti et al., 2008; Fairhurst et al., 2013), would be associated with reduced body condition (Fairhurst et al., 2013; Rubolini et al., 2005; Wada & Breuner, 2010). Indeed, more optimal parental provisioning rates have earlier been suggested to reduce circulating CORT levels (Kitaysky et al., 2001b), which explains the general finding that nestlings in good nutritional condition had lower CORT_f_ levels (e.g., Lopez‐Jimenez et al., 2016; Will et al., 2014). However, in our study, factors that influenced CORT_f_ and SMI likely operated over different periods. CORT_f_ values reflect circulating CORT levels over the entire feather growth period. Hence, especially in nests with large broods and/or those that suffered brood reduction, this includes the period of strong nestling competition and the period following brood reduction. By contrast, SMI was computed from metrics scored at the end of the nestling period and may therefore provide information over a relatively limited period of time compared to feather growth. Early in the nestling period, in a competitive brood environment, higher CORT levels may actually have benefitted those nestlings in terms of begging and/or positioning close to the nest hole to obtain more food (e.g., Loiseau, Sorci, Dano, & Chastel, 2008a; Ruppli et al., 2012) and allowed them to survive the brood reduction process. After brood reduction, sibling competition for food was probably relaxed among the remaining nestlings, resulting in a high body condition at the end of the nestling period, but concurrent reductions in circulating CORT may not have been reflected in CORT_f_ because levels were low for a short period in time relative to the entire feather growth period (Romero & Fairhurst, 2016). Such a scenario could explain higher CORT_f_ in nests that suffered brood reduction and also explain a positive association between CORT_f_ and SMI. Increased CORT levels early in the nestling period could, however, come at the cost of an unstable hormonal/developmental setting and thus increased levels of FA.

In addition, recent studies suggested that interpreting CORT_f_ levels can be complex because variations in feather density may exert an important effect on CORT deposition (Patterson, Kitaysky, Lyons, & Roby, 2015). Given that CORT is passively deposited during feather formation (Bortolotti, 2010; Jenni‐Eiermann et al., 2015; Romero & Fairhurst, 2016), it is possible that CORT_f_ is influenced by the amount of material incorporated into growing feathers, resulting in lower CORT_f_ in less dense feathers, regardless of circulating CORT levels (Patterson et al., 2015). Both high plasma CORT concentrations and food limitation can interfere with feather development and feather quality, resulting in feathers that are lighter, weaker and with an altered microstructure (DesRochers et al., 2009; Lattin, Reed, DesRochers, & Romero, 2011). So, even if nutritional stress and decreased body condition typically increase plasma CORT (e.g., Kitaysky et al., 2001b; Müller, Jenni‐Eiermann, & Jenni, 2010; Poisbleau, Demongin, Chastel, Eens, & Quillfeldt, 2010), concurrent CORT_f_ elevations should only be expected if feather quality remains unaffected by nutritional status (Lopez‐Jimenez et al., 2016; Patterson et al., 2015; Will et al., 2014; see Romero & Fairhurst, 2016 for a review). To our knowledge, no study has examined how nutritional condition affects CORT_f_ in relation to feather development and quality in passerine species, and so experimental validation is needed to reliably interpret CORT in passerine nestling feathers.

In conclusion, it seems that food supplementation did not affect indices of nestling quality directly. However, early in the breeding season, nestlings from food supplemented nests did seem to suffer from more stressors, potentially from increased sibling competition, that were absent in control nests or in nests initiated later in the breeding season. Although experimental validation is needed to know how nutritional status affects development and quality of house sparrow traits, our study provides support for the hypothesis that food supply during early life has implications for nestling quality. Yet, the observed inter‐relationships among‐nestling SMI, tarsus‐based FA and CORT_f_ all suggest that any mechanism linking food supply to population growth in this species remains highly complex.

## CONFLICT OF INTEREST

None declared.
