# Supplementary material for: Supplementary feeding increases nestling feather corticosterone early in the breeding season in house sparrows
Source: Ecol Evol. 2017 Jun 30;7(16):6163–71. doi: 10.1002/ece3.3114 (PMC5574790; doi:10.1002/ece3.3114)

**Supplementary Material**

**Table S1.** Effect of mealworm provision on the average number of fledglings produced from individual nesting attempts of house sparrows in suburban-rural Leicester in 2008. The difference in fledgling production between fed and unfed nests was statistically significant (Wald test: *F*_1,60_ = 9.68, *P*<0.003) after allowing for any effects of study area (six localities), landscape (suburban, rural) and month when first egg was laid (May, June, July). Adapted from Peach et al. (2014)

_____________________________________________________

Treatment Number of Mean (SE) number
 nests of fledglings per nesting attempt

_____________________________________________________

Fed 34 2.79 (0.230)

Unfed 91 1.80 (0.148)

_____________________________________________________

**Table S2.** Results from all explanatory variables tested to explain variation in CORT_f_ levels. P-values of the reduced model are shown in bold, while results from all other variables were obtained by adding them one at a time onto the reduced model.


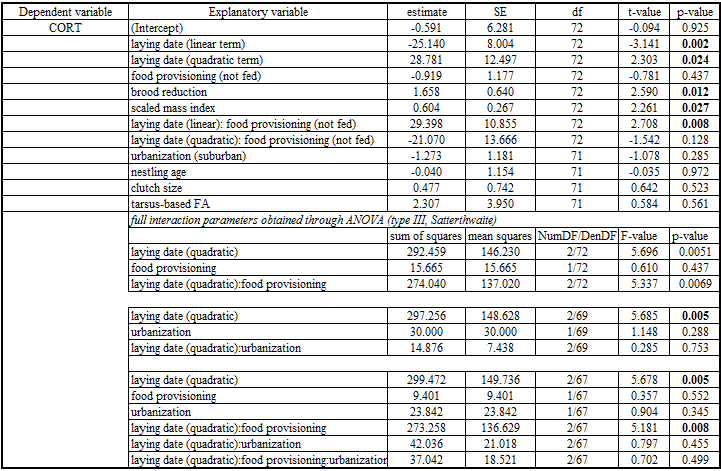


Note that for the three stress indicators (CORT-levels, tarsus-based FA and SMI), models were run with laying date as a linear or a quadratic term (see main text). For CORT-levels, the model with laying date as quadratic term performed best (see main text), and this quadratic model is therefore presented here.

**Table S3.** Results from all explanatory variables tested to explain variation in Tarsus-based FA. P-values of the reduced model are shown in bold, while results from all other variables were obtained by adding them one at a time onto the reduced model.


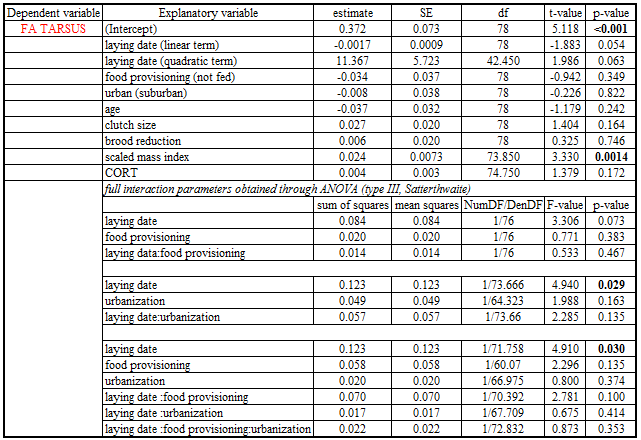


**Table S4.** Results from all explanatory variables tested to explain variation in SMI (Scaled Mass Index). P-values of the reduced model are shown in bold, while results from all other variables were obtained by adding them one at a time onto the reduced model.


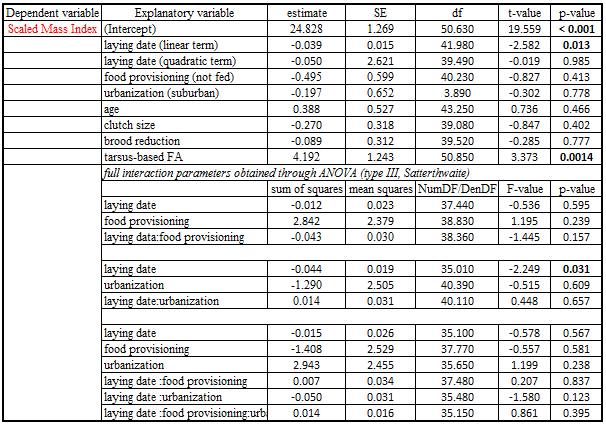


**Table S5.** Results from all explanatory variables tested to explain variation in the range of CORT_f_ levels per house sparrow nest. P-values of the reduced model are shown in bold, while results from all other variables were obtained by adding them one at a time onto the reduced model.


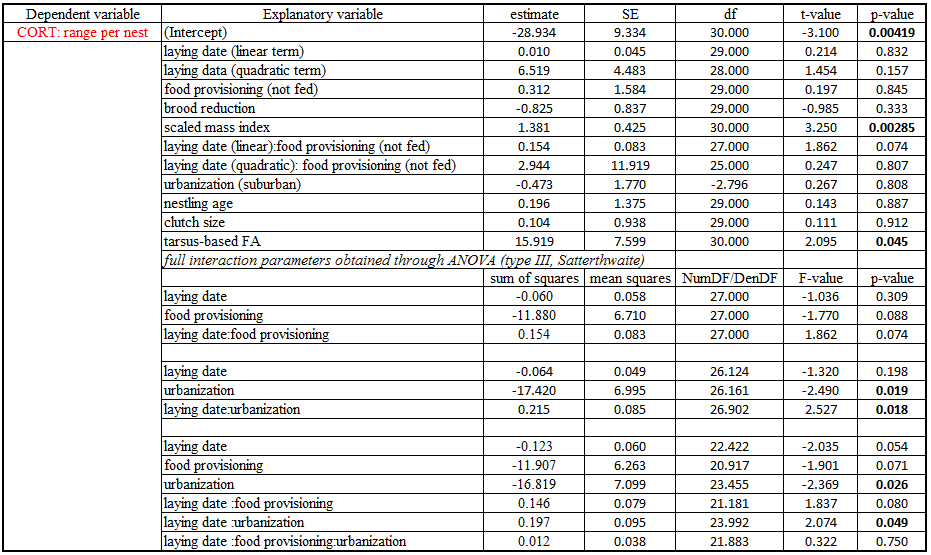


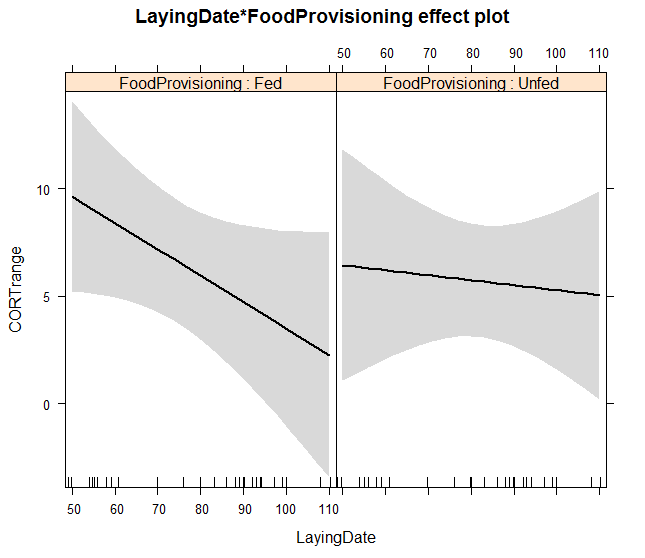


**Table S6.** Results from all explanatory variables tested to explain variation in the range of tarsus-based FA values per house sparrow nest. P-values of the reduced model are shown in bold, while results from all other variables were obtained by adding them one at a time onto the reduced model.


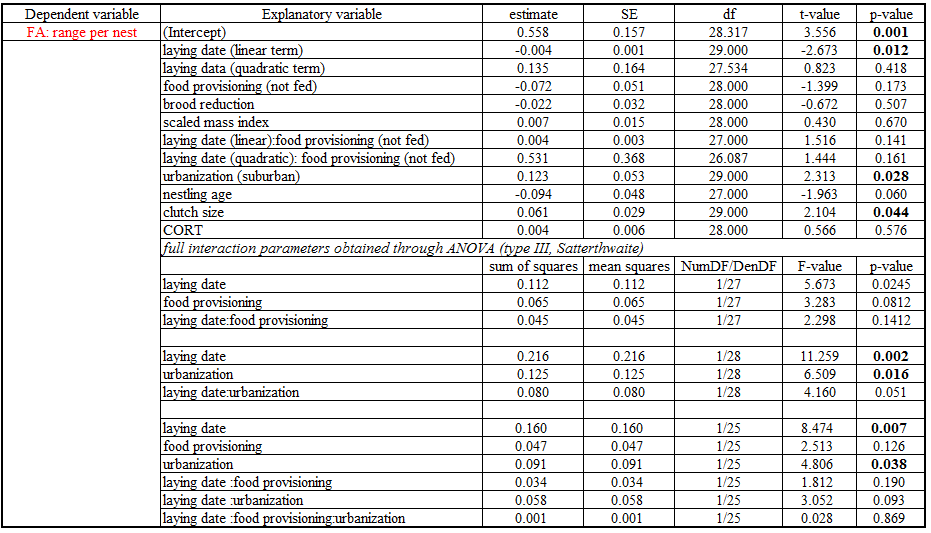


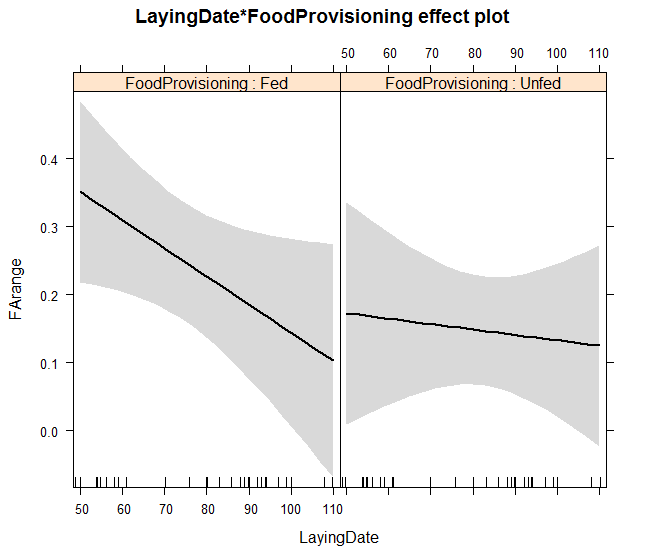


**Table S7.** Results from all explanatory variables tested to explain variation in the range of SMI (Scaled Mass Index) per house sparrow nest. P-values of the reduced model are shown in bold, while results from all other variables were obtained by adding them one at a time onto the reduced model.


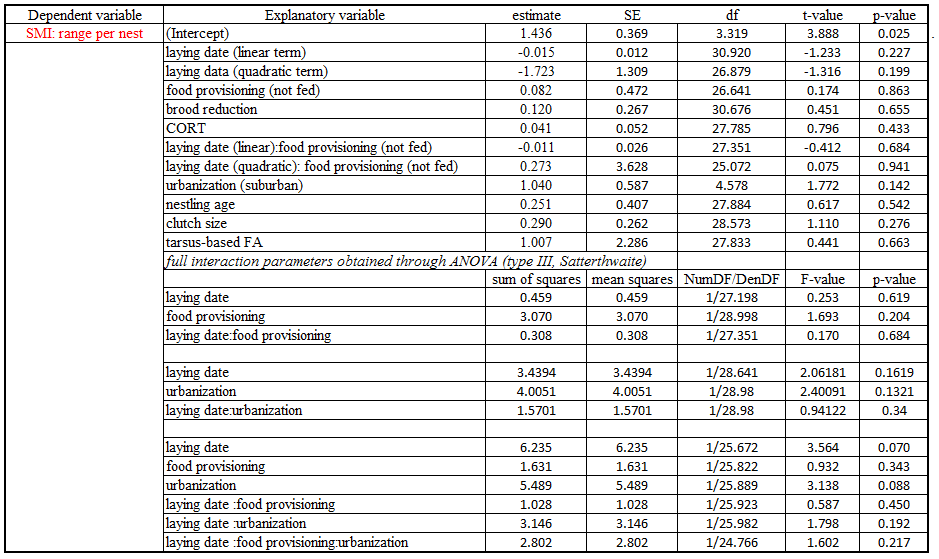


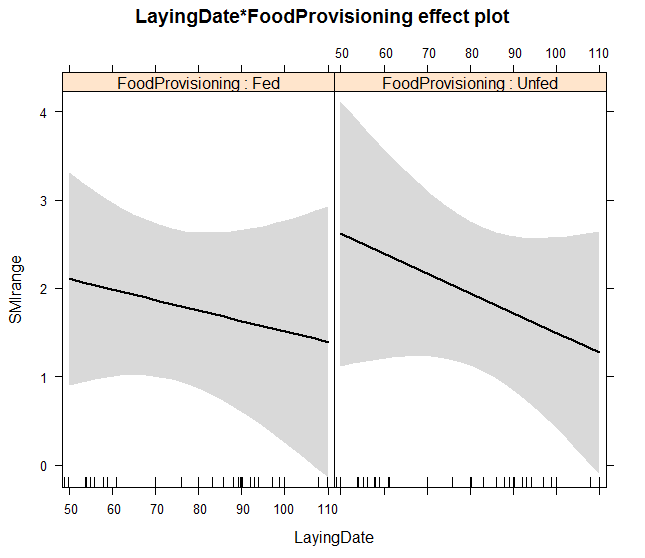

Supplement: Supplementary file 1 [file ECE3-7-6163-s001.doc]
